# Fluid administration rate for uncontrolled intraabdominal hemorrhage in swine

**DOI:** 10.1371/journal.pone.0207708

**Published:** 2018-11-29

**Authors:** Ujwal R. Yanala, Jason M. Johanning, Iraklis I. Pipinos, Robin R. High, Gustavo Larsen, William H. Velander, Mark A. Carlson

**Affiliations:** 1 Department of Surgery, University of Nebraska Medical Center, Omaha, Nebraska, United States of America; 2 Department of Surgery, VA Nebraska–Western Iowa Health Care System, Omaha, Nebraska, United States of America; 3 Department of Biostatistics, University of Nebraska Medical Center, Omaha, Nebraska, United States of America; 4 Department of Chemical and Biomolecular Engineering, College of Engineering, University of Nebraska–Lincoln, Lincoln, Nebraska, United States of America; 5 Department of Genetics, Cell Biology and Anatomy, University of Nebraska Medical Center, Omaha, Nebraska, United States of America; Medical Center of the Johannes Gutenberg-University, GERMANY

## Abstract

**Background:**

We hypothesized that slow crystalloid resuscitation would result in less blood loss and a smaller hemoglobin decrease compared to a rapid resuscitation during uncontrolled hemorrhage.

**Methods:**

Anesthetized, splenectomized domestic swine underwent hepatic lobar hemitransection. Lactated Ringers was given at 150 or 20 mL/min IV (rapid *vs*. slow, respectively, N = 12 per group; limit of 100 mL/kg). Primary endpoints were blood loss and serum hemoglobin; secondary endpoints included survival, vital signs, coagulation parameters, and blood gases.

**Results:**

The slow group had a less blood loss (1.6 *vs*. 2.7 L, respectively) and a higher final hemoglobin concentration (6.0 *vs*. 3.4 g/dL).

**Conclusions:**

Using a fixed volume of crystalloid resuscitation in this porcine model of uncontrolled intraabdominal hemorrhage, a slow IV infusion rate produced less blood loss and a smaller hemoglobin decrease compared to rapid infusion.

## Introduction

Massive hemorrhage and traumatic brain injury each account for about half of early mortality on the modern battlefield.[1, 2] Preclinical and clinical research directed at these difficult scenarios has evolved management strategies, such as such as rapid evacuation and damage control resuscitation, which have improved outcomes for critically injured personnel.[3, 4] A central tenant of damage control resuscitation has been the resuscitation of a casualty in hemorrhagic shock with whole blood (preferred) or with blood products using a 1:1:1 ratio of plasma, red blood cells, and platelets.[5] If blood products are not available, then colloid and/or crystalloid fluids are given in 500 mL boluses “until a palpable radial pulse, improved mental status or systolic BP of 80–90 mmHg is present.”[5] This latter aspect of damage control resuscitation, in which crystalloid administration is minimized during the transport phase of a subject with hemorrhagic shock until the subject reaches a forward surgical unit, also has been described as hypotensive resuscitation.[4, 6, 7]

Avoidance of aggressive fluid resuscitation in trauma victims prior to surgical hemostasis was advocated as early as World War I.[8] In the modern era, animal[6, 9, 10] and clinical[3, 7, 11–13] data have indicated that hypotensive resuscitation improves survival after severe hemorrhagic injury. As a result, the basic tenants of hypotensive resuscitation have been adopted[4] into the Tactical Combat Casualty Care (TCCC) Guidelines of the United States Department of Defense. The current TCCC Guidelines[5] do not explicitly state parameters for either a fluid maximum or an optimal fluid administration rate with respect to the prehospital management of a victim with uncontrolled hemorrhage and shock. The topic of the present study was the issue of optimal fluid administration rate. We hypothesized that for a fixed volume of resuscitation fluid in a porcine model of uncontrolled intraabdominal hemorrhage, a slow rate of intravenous infusion would result in less blood loss and a smaller hemoglobin decrease compared to a more rapid rate.

## Materials and methods

### Animal welfare

Refer to the ARRIVE (Animal Research: Reporting of *In Vivo* Experiments[14]) information in S1 Table. This animal research study was carried out in accordance with recommendations in the *Guide for the Care and Use of Laboratory Animals* (8^th^ ed.) from the National Research Council and the National Institutes of Health,[15] and also in accordance with the Animal Welfare Act of the United States (U.S. Code 7, Sections 2131–2159). The animal protocol was approved by the Institutional Animal Care and Use Committee (IACUC) of the VA Nebraska-Western Iowa Health Care System (protocol number 00760), by the IACUC of the University of Nebraska Medical Center (protocol number 11-064-07-ET), and by the Animal Care and Use Review Office (ACURO) of the United States Army Medical Research and Materiel Command (award number W81XWH-11-1-0836). All procedures were performed in animal facilities approved by the Association for Assessment and Accreditation of Laboratory Animal Care International (AAALAC; http://www.aaalac.org) and by the Office of Laboratory Animal Welfare of the Public Health Service (http://grants.nih.gov/grants/olaw/olaw.htm). All surgical procedures were performed under isoflurane anesthesia, and all efforts were made to minimize suffering. Euthanasia was performed in accordance with the AVMA Guidelines for the Euthanasia of Animals.[16]

### Study design and determination of subject numbers

The study design of this report was a non-randomized case-control type (see Discussion). The minimum number of swine (n = 12) utilized in each group was determined with a statistical power analysis[17] using Δ/σ (Cohen's *d*, in which Δ is the desired difference in means of numerical data set by the observer, and σ is the estimated standard deviation) = 1.25, false positive rate (α) = 0.05, false negative rate (β) = 0.2, and power (1 – β) = 0.8. The endpoints targeted in the power analysis were blood loss and final hemoglobin level.

### Animal preparation

**Fig 1:**
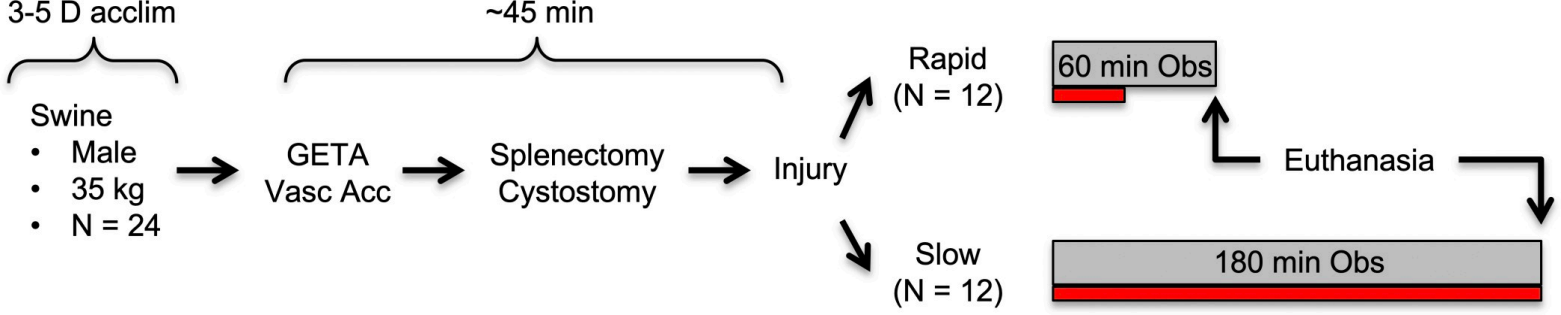
Experiment flow chart. Swine were acclimatized (acclim), then on the procedure day underwent general endotracheal anesthesia (GETA), followed by access of the carotid and jugular vessels (Vasc Acc), and then other preparatory procedures. After injury, subjects underwent IV crystalloid resuscitation (maximum volume allowed = 100 mL/kg) for the indicated observation period (Obs). Red bar underneath each observation period indicates relative time required for crystalloid infusion.

Refer to the flow diagram in Fig 1. Domestic swine (castrated males, 3 months) were purchased from the Agricultural Research and Development Center (Mead, NE) of the University of Nebraska–Lincoln, and acclimatized for 3–5 days under veterinary supervision. Subjects were fed *ad libitum* with corn-soybean meal and water. Subjects were fasted for 12 h prior to surgery, but with no water restriction. Immediately prior to the procedure, animals were premedicated[18] with a single 3 mL IM injection containing 150 mg Telazol (tiletamine hydrochloride and zolazepam hydrochloride, 1:1 by weight; Fort Dodge Animal Health, New York, NY), 90 mg ketamine, and 90 mg xylazine.

Sedated subjects then were weighed, intravenous access was established via an ear vein, endotracheal intubation was performed, and general anesthesia was maintained with 1–2% isoflurane throughout the procedure using a Matrx Model 3000 Veterinary Anesthesia Ventilator (Midmark Corp., Versailles, OH).[19] Central arterial and venous lines were placed through a cutdown in the right neck for pressure monitoring, blood sampling and fluid resuscitation. MAP (mean arterial pressure), end-tidal pCO2, rectal temperature, cardiac electrical activity, and pulse oximetry were continuously recorded.[20] Mechanical ventilation was maintained at 12–15 breaths per minute, with a tidal volume of 10–15 mL/kg, in order to keep the end-tidal pCO_2_ at 35–45 mm Hg.[20, 21] Since hypothermia was not an intended variable in this study, a water-circulated warming pad (set at 39°C) was placed under each subject to support body temperature.[22, 23]

A ventral midline laparotomy incision was made, splenectomy was performed,[6, 10, 24–27] and a transabdominal cystostomy tube was placed.[20] Per published protocols,[24, 25, 27] the excised spleen was weighed and then a volume of warm lactated Ringers (LR; 37°C) solution equivalent to three-fold the splenic weight was administered through the jugular line, using a rapid infusion pump (Cole-Palmer Masterflex L/S; Vernon Hills, IL) set at 100 mL/min. Prior to injury, any blood loss incurred during the preparation was quantified by weighing tared surgical sponges that were used to absorb lost blood, and then a volume of LR equivalent to three-fold the pre-injury blood loss (typically <50 mL) was given using the infusion pump.

### Injury mechanism, resuscitation, and observation

Pre-injury vital signs were recorded, the lower half of the midline incision was closed with towel clips, and then the injury mechanism (hepatic left lower lobe hemitransection) was applied, as previously described (a 4 cm cut across the base of the left lateral lobe of the liver[20]), producing a combined portohepatic venous injury. Immediately after injury, the laparotomy incision was closed with towel clips. All the subjects were allowed to bleed without any efforts at local hemostasis (compression, bandage, vessel clamping, etc.).

The goal of the post-injury resuscitation was to give all subjects the same volume of fluid, but with two different administration rates. When the subject’s post-injury MAP dropped below 80% of the pre-injury MAP (defined as the target MAP[20, 26, 28, 29]), LR solution (stored at 37°C) was begun at either 150 or 20 mL/min IV (rapid and slow group, respectively, N = 12 per group) using the infusion pump. LR was selected as the resuscitation fluid secondary to its use in multiple previous investigations that (*i*) utilized porcine hemorrhage models and (*ii*) had military relevance.[20, 26, 28, 29] The maximum volume of post-injury LR resuscitation was capped at 100 mL/kg.[20] Resuscitation fluid was administered as long as the MAP was below the target level,[20, 21, 26, 28] until the animal expired, or until the 100 mL/kg fluid maximum was reached. Similar resuscitation regimens using similar volumes of crystalloid have been utilized in various porcine hemorrhage models by other author groups.[21, 26, 28, 30–32]

The maximum post-injury observation time for the rapid *vs*. slow groups was 60 *vs*. 180 min, respectively. Subjects remained under general anesthesia, with continuous monitoring of vital signs and periodic blood draws for laboratory testing. If a subject was still alive at the end of the prescribed observation period, it was euthanized by increasing the isoflurane fraction to 5% for 5 minutes, followed by bilateral diaphragm incision with transection of the supradiaphragmatic inferior vena cava (intentional exsanguination as approved by the AMVA[16]).

### Endpoints

Primary endpoints were blood loss and serum hemoglobin; secondary endpoints included survival, vital signs, coagulation parameters, and blood gases. Heart rate, MAP, pulse oximetry, end-tidal pCO2, and rectal temperature were continuously recorded, as described above. Blood samples from an arterial line were drawn pre-injury, at 15 min post-injury, and then at the “final” time point, which was defined as the time of imminent death (for those subjects not surviving the prescribed observation period) or upon completion of the prescribed observation period (60 min in the rapid infusion group, and 180 min in the slow infusion group). Each set of laboratory tests included arterial blood gases, blood count, and coagulation testing. Imminent death was defined as MAP ≤20 mm Hg with a pulse pressure of ≤10 mm Hg. Death was defined as MAP ≤20 mm Hg with no identifiable pulse pressure on the monitor’s arterial tracing, end-tidal pCO_2_ <5 mm Hg, and absent corneal reflex.

Immediately after each subject was declared dead, the laparotomy incision was re-opened, and all clots and blood were rapidly evacuated using a combination of tared laparotomy pads, suction, and manual extraction. Tared buckets containing evacuated blood were weighed in order to calculate blood loss. Necropsy was performed immediately after expiration; the liver was explanted for inspection, dissection, photography, and documentation of the injury anatomy.

The complete blood count, prothrombin time (PT), activated partial thromboplastin time (APTT), international normalized ratio (INR), quantitative fibrinogen assay (QFA), and arterial blood gas (ABG) testing were contracted to the Clinical Laboratory of the VA Nebraska-Western Iowa Health Care System. This laboratory used the von Clauss method for the QFA.

### Statistical analysis

Numerical data were reported as the mean ± standard deviation (SD). For a complete description of the statistical analysis, see S1 Fig. Unpaired continuous data were compared with ANOVA; the level of significance was defined as p < 0.05. Groups of categorical data were compared with the Fisher exact test. If data for a given subject at a given time point were “missing” (e.g., no data captured secondary to lost blood tube, clotting of a blood specimen prior to running an assay, monitor malfunction, other miscellaneous events), then the respective cells in the data spreadsheet were kept empty (see S2 Table). If a coagulation test was reported as “failed” (meaning, no clot formation during the assay), then the respective data cells were filled as follows: QFA = 20 mg/dL; PT = 37 s; INR = 5 (critical values from contracting laboratory).

## Results

### Pre-injury data

All raw data from this study and a detailed statistical analysis thereof are contained in S2 Table and S1 Fig, respectively. Mean subject weights were 35.8 ± 1.8 and 37.9 ± 4.9 kg, rapid *vs*. slow groups, respectively (p = 0.181, unpaired t-test). Pre-injury blood loss (incurred during subject preparation, and including splenic mass) was not different between the two groups (Table 1); splenic mass was 360 ± 71 *vs*. 343 ± 75 g, rapid *vs*. slow groups, respectively (p = 0.583, unpaired t-test). Pre-injury fluid administration, which included splenic replacement per the 3x formula described in the Methods, also was not different between the two groups (Table 1). The pre-injury MAP, heart rate, temperature, hemoglobin concentration, platelet count, QFA, PT, and ABG data were not different between two groups (Tables 2–5; see also S1 Fig). There was a small but significant difference in the INR values between the two groups (Table 4), but this did not seem to be physiologically relevant.

**Table 1:** Blood loss, fluid input, liver weight, and lacerated veins.

	Blood Loss (mL)*		Fluid input (mL)*			No. Veins Lacerated*	
Infusion Rate	Pre-injury	Post-injury	Pre-injury	Post-injury	Liver weight (g)*	Hepatic	Portal
Rapid	430 ± 151	2738 ± 693	1288 ± 458	3644 ± 523	852.9 ± 114.8	0.9 ± 0.3	1.3 ± 0.4
Slow	414 ± 48	1600 ± 360	1138 ± 181	3082 ± 1720	941.0 ± 127.3	1.0 ± 0.0	1.4 ± 0.5
p-value	0.72	**<0.001**	0.31	0.30	0.089	0.4884	0.7075

All values are mean ± SD; time points are with respect to injury

*unpaired t-test (significant values are bolded).

**Table 2:** Vital sign data.

	MAP (mm Hg)			Heart rate (beats/min)			Temperature (°C)		
Infusion Rate	Initial	15 min	Final	Initial	15 min	Final	Initial	15 min	Final
Rapid	113 ± 14	55 ± 31	27 ± 16	107 ± 21	108 ± 22	100 ± 23	36.9 ± 0.6	36.5 ± 0.5	36.0 ± 0.6
Slow	105 ± 18	33 ± 17	39 ± 31	90 ± 19	110 ± 25	112 ± 23	36.3 ± 1.1	35.9 ± 1.0	35.1 ± 1.0
*p-value	0.52	0.11	0.48	0.09	0.99	0.69	0.23	0.15	**0.010**

MAP = Mean Arterial Pressure. All values are mean ± SD; time points are with respect to injury

*unpaired t-test; significant tests are bolded.

**Table 3:** Hematologic testing results.

	Hemoglobin (g/dL)			Platelets (x 1,000/μL)		
Infusion Rate	0 min	15 min	Final	0 min	15 min	Final
Rapid	12.2 ± 0.9	4.6 ± 2.0	3.4 ± 2.3	306 ± 77	147 ± 66	122 ± 66
Slow	11.7 ± 0.9	8.4 ± 1.2	6.0 ± 2.2	315 ± 87	215 ± 55	171 ± 71
p-value*	0.39	**<0.001**	**0.025**	0.99	**0.029**	0.23

All values are mean ± SD; time points are with respect to injury

*unpaired t-test; significant values are bolded.

**Table 4:** Coagulation testing results.

	QFA (mg/dL)			Protime (s)			INR		
Infusion Rate	0 min	15 min	Final	0 min	15 min	Final	0 min	15 min	Final
Rapid	115 ± 13	41 ± 12	37 ± 13	11.5 ± 0.7	16.6 ± 4.7	18.9 ± 8.9	1.0 ± 0.1	1.5 ± 0.5	2.0 ± 1.3
Slow	109 ± 17	63 ± 9	47 ± 15	10.9 ± 0.6	11.3 ± 1.4	14.4 ± 9.5	0.9 ± 0.1	1.0 ± 0.1	1.4 ± 1.5
p-value*	0.58	**<0.001**	0.27	0.11	**0.003**	0.46	**<0.001**	**0.004**	0.69

QFA = Quantitative Fibrinogen Assay; INR = International Normalized Ratio. All values are mean ± SD; time points are with respect to injury

*unpaired t-test; significant values are bolded.

**Table 5:** Arterial blood gas results.

	Rapid (N = 12)		Slow (N = 12)		
ABG parameter	Mean	SD	Mean	SD	p-value*
pH, initial	7.46	0.07	7.44	0.10	0.659
pH, 15 min	7.40	0.10	7.44	0.09	0.438
pH, final	7.45	0.10	7.37	0.18	0.237
pCO2 (mm Hg), initial	39.3	7.1	46.5	11.6	0.085
pCO2 (mm Hg), 15 min	36.5	12.7	34.4	10.8	0.671
pCO2 (mm Hg), final	32.1	15.4	34.6	16.6	0.709
pO2 (mm Hg), initial	443	27	451	34	0.548
pO2 (mm Hg), 15 min	445	34	434	50	0.561
pO2 (mm Hg), final	414	48	414	55	0.983
Bicarbonate (mmol/L), initial	3.4	2.5	4.4	2.6	0.315
Bicarbonate (mmol/L), 15 min	-2.8	4.2	-1.7	2.4	0.454
Bicarbonate (mmol/L), final	-3.4	6.7	-6.4	6.2	0.281
Base Excess (mmol/L), initial	27.0	2.5	28.7	2.3	0.096
Base Excess (mmol/L), 15 min	21.5	4.3	21.8	3.4	0.864
Base Excess (mmol/L), final	20.7	6.7	18.3	5.8	0.391

Final value was obtained at imminent death or at completion of observation period (60 or 180 min for the Rapid vs. Slow group, respectively).

*Unpaired t-test.

### Primary endpoints: Blood loss and hematology data

Post-injury blood loss was ~70% greater in the rapid group compared to the slow group (~2.7 *vs*. 1.6 L, respectively), but total post-injury crystalloid administered was not different (Table 1). The latter result was intentional per the experimental design; i.e., the total maximum crystalloid volume was set at 100 mL/kg for a subject in either group. Post-injury serum hemoglobin in the rapid group was about half the value of the slow group at both the 15 min and final time points; platelet concentration was lower in the rapid group at the 15 min time point only (Table 3). The QFA, PT, and INR were all more negatively affected in the rapid group compared to the slow group at the 15 min time point (Table 4). Data on APTT were not analyzed secondary to an excessive number of missing values.

### Injury survival

For a full description of the typical response to this injury, including video, please refer to a previous publication.[20] The survival of the rapid *vs*. slow infusion groups was 58% (7/12 subjects) *vs*. 67% (8/12 subjects), respectively; p = 0.500 (Fisher exact test; see Kaplan-Meier plot in S2 Fig). The five subjects in the rapid group that expired prior to the end of the prescribed observation period were pronounced dead at 15, 26, 38, 40, and 43 min after injury; the four subjects in the slow group that similarly expired died at 30, 34, 35, and 53 min after injury. All deaths occurring prior to the prescribed observation period were preceded by terminal hypotension (i.e., no dysrhythmia, ventilatory inadequacy, or other contributing factors were noted).

### Vital sign data

The MAP dropped precipitously (from >100 mm Hg to 30–60 mm Hg) within each group by 1–2 min after injury, but there were no significant differences in post-injury MAP between the rapid and slow groups (Table 2) at the 15 min or final time points. Interestingly, the MAP in the rapid group trended higher (nonsignificant) at the 15 min time point with respect to the slow group, but the opposite nonsignificant trend was present at the final time point (i.e., the MAP of the slow group trended higher). Although there was a small significant difference (~1°C) in temperature at the final time point, there were no post-injury differences between the two groups for heart rate and temperature (Table 2).

### Blood gas and necropsy results

The within-group pH, pCO_2_, bicarbonate, and base excess all trended down after injury, but there were no differences in these values comparing the rapid *vs*. slow groups at any time point (see Table 5, and also S1 Fig). End-tidal pCO_2_ was monitored but the electronic readout was not reliably captured, so comparative analysis of these data were not possible. Liver mass at necropsy not different between the groups (Table 1). Postmortem dissection of the explanted liver demonstrated that there was no difference between the two groups with respect to the number of hepatic or portal veins that were lacerated (Table 1).

## Discussion

In this study of uncontrolled intraabdominal hemorrhage from a combined portohepatic venous injury in swine, it was found that for a fixed volume of resuscitation fluid, a 20 mL/min intravenous infusion rate (“slow”) resulted in a reduction of blood loss and less derangement of hemoglobin, platelet, and coagulation parameters compared to an infusion rate of 150 mL/min (“rapid”). The actual volume of post-injury crystalloid given did not differ between the two groups (this volume was capped at 100 mL/kg). So, what was really tested in this study was the effect of the pump rate for a fixed volume of fluid. Essentially, subjects with the slower pump rate did better.

Although there was less blood loss and a smaller hemoglobin decrease (primary endpoints) in the slow group compared to the rapid group, the final mean temperature (a secondary endpoint) was about 1°C greater in the rapid infusion group. This result likely was secondary to the fact that rapid group received the fixed volume of pre-warmed LR resuscitation fluid over a shorter time period. It is not clear whether the difference in final rectal temperature (36 *vs*. 35°C) was biologically relevant.

A ready explanation for the apparent detrimental effect of rapid intravenous resuscitation for hemorrhagic shock seen in this study was hemodilution. Although both groups received the same fluid volume of crystalloid in the post-injury period, perhaps the slower infusion rate provided adequate time for that infused fluid to equilibrate into the extravascular space without causing hemodilution, while the rapid infusion rate overwhelmed the vascular space and diluted its contents. Furthermore, it has been demonstrated in animal models that saline infusion can activate systemic inflammation;[33, 34] what role this phenomenon may have played in the present study was not determined.

Beyond hemodilution and activation of systemic inflammation, other possible explanations for the finding that a slower infusion rate resulted in less blood loss and a higher final hemoglobin concentration than the rapid infusion rate include: (1) an inhibitory effect of rapid crystalloid infusion on the coagulation cascade (i.e., beyond the effect of simple hemodilution of the clotting factors); (2) an inhibitory effect of rapid crystalloid infusion on platelet function or quantity (beyond the effect of hemodilution of platelet number); (3) momentary surges in blood pressure that might occur with rapid crystalloid infusion, which might disrupt the adhesion of fresh clot at a hemorrhage site; and (4) inhibition of clot cross-linking at the injury site, which would decrease the effectiveness of clot adhesion. Needless to say, these possibilities are all hypotheses that have not been examined at this point.

This study does suggest that there may be a dose-response effect of resuscitation fluid infusion rate in the setting of exsanguinating intraabdominal hemorrhage. However, we as yet do not have sufficient data in swine to specify a cut-off of “too fast” or “too slow” with regard to this infusion rate. Whether subjects with exsanguinating hemorrhage in the field should be given any prehospital crystalloid resuscitation at all is a provocative question, but one that cannot be answered at present. Most available data suggest that prehospital resuscitation using plasma or whole blood in subjects with exsanguinating hemorrhage is preferable to crystalloid,[35–37] though this conclusion was not reached in a recent trial comparing prehospital plasma to saline resuscitation in a study of urban trauma patients.[38]

Parameters describing a fluid maximum or fluid administration rate for prehospital crystalloid administration are not described in the current TCCC Guidelines.[5] While this study cannot provide these parameters, the data contained in this report can contribute to the ongoing discussion on how this aspect of prehospital management might evolve. In particular, it may ultimately be determined that a relatively slow intravenous infusion rate (as opposed to bolus infusion described in the current TCCC Guidelines[5]) may be preferred for prehospital fluid resuscitation of the patient with hemorrhagic shock for which blood products are not immediately available.

Overall, the preclinical and clinical data support the use of low-volume resuscitation for prehospital management of hemorrhagic shock. The present study supports using a slow infusion rate as opposed to rapid infusion for a relatively large (100 mL/kg) fixed volume of crystalloid resuscitation. Whether additional benefit might be gained by combining a slow infusion rate with a low fixed volume of resuscitation fluid will require additional study.

### Previous Studies

In previous studies, 2-h survival in non-resuscitated swine with uncontrolled aortic hemorrhage was 100%, while it was <50% in subjects resuscitated with bolused hypertonic saline/dextran (4 mL/kg over 1 min) or rapidly infused lactated Ringer’s (80 mL/kg over 9 min).[9] Swine undergoing a 40% blood volume bleed through a carotid arterial catheter and not resuscitated had somewhat better coagulation parameters after a 2-h observation period compared to swine who were fluid resuscitated.[39] Swine undergoing a grade V liver injury and not resuscitated shed less blood compared to fluid resuscitated swine after a 2-h observation period,[40] though blood pressure was marginally less in the former swine. In a combined femoral/aortic hemorrhage model in swine, resuscitation to a MAP of 40 mm Hg produced better 1-h survival than a resuscitation target of 80 mm Hg or no resuscitation at all.[6]

Regarding previous preclinical studies which specifically evaluated the effect of fluid administration rate on hemorrhagic shock, less data is available. Sondeen *et al*. found that the rate of the lactated Ringer’s resuscitation (100 *vs*. 300 mL/min) did not affect the time at which swine rebled from an aortotomy;[22] the major determinant of rebleeding (their primary endpoint) was a MAP greater than ~60 mm Hg. Swine resuscitated with the 300 mL/min rate in this study, however, had a lower serum hemoglobin at the point of rebleeding. In regards to clinical studies, reports since the 1990’s have suggested[3, 7, 11–13] that if blood products are not available, then fluid restriction (i.e., hypotensive resuscitation) in the pre-hospital management of hemorrhagic shock may produce better outcomes.

We utilized routine pre-injury splenectomy in this study based on the published experience of multiple groups in this field, which has described an auto-transfusion capability of the spleen during hemorrhagic shock.[6, 10, 24–27] We also have observed this auto-transfusion phenomenon ourselves; after shock-inducing injury, the porcine spleen can contract to ~one-tenth of its original volume (unpublished observations). We do acknowledge, however, that splenectomy for prevention of splenic autotransfusion in porcine hemorrhage models is controversial.[41, 42]

### Study limitations

The subjects of this study were not randomized, because this study was not a pre-planned trial in the authors’ laboratory. Our primary task had been to develop hemostatic technologies for uncontrollable intraabdominal hemorrhage (unpublished data) using a porcine model,[20] in which we utilized a rapid fluid-resuscitation protocol frequently used in these models.[21, 26, 28, 30–32] Since this protocol was not completely consistent with the TCCC Guidelines,[5] and because we believed that rapid fluid resuscitation was negatively affecting our outcomes, we switched to a slower fluid resuscitation rate, but using the same total volume limit. Our subsequent impression was that the swine were doing better with the slower rate. At that point we decided to perform a comparison of the fast vs. slow resuscitation groups, which thus produced the case-control design of this manuscript.

This experimental design (case-control) is a flaw of this study; the experimental groups are not strictly comparable. For example, the slow resuscitation protocol required extending the post-injury observation period to 180 min (compared to the 60 min observation period of the fast group), so that the total resuscitation volume in the fast *vs*. slow groups would be equal. However, despite the slow group having three times the duration of post-injury observation, this group still lost ~1 L less blood than the rapid group. We believe that this is a significant result that is not negated by the study design.

We could have compared the two resuscitation protocols at one hour, thus eliminating the issue of differing durations of post-injury monitoring, but then the total fluid volume would not have been equal between groups. Alternatively, we could have redone the study in a randomized fashion, using a 3 h observation period in all subjects. But given the convincing outcomes obtained with the case-control subjects reported herein, we decided not to proceed with a formal trial, because we did not believe it would be ethical in regards to animal utilization. That is, we thought that a formal controlled trial that studied fluid resuscitation rates would be unnecessary, because of the existing data. Another potential issue with this study is the lack of a no-treatment control group, which has been utilized in other preclinical studies of fluid resuscitation in hemorrhagic shock.[6, 9, 39, 40] Given the fact that the current TCCC Guidelines[5] do not recommend non-resuscitation in the prehospital phase, we decided not to include a no-treatment group in our study.

With 12 subjects per group, this study was not powered to detect a difference in survival (a secondary endpoint) between the rapid *vs*. slow crystalloid infusion groups. If the survival of the rapid infusion group is held constant at 58%, then in order to have 80% power to detect a significant difference (p < 0.05) with the Fisher exact test, the survival of the slow infusion group would need to be 100% with 17 subjects per group. Alternatively, in order to detect a survival difference of 20% at 80% power, the study would have required 85 subjects per group. So, with only 12 subjects per group, the ability of the present study to detect a difference in survival was limited.

In addition to the above issues, other limitations of this study include: no long-term survival data, no long-term data on organ dysfunction; no histological data on acute organ injury; no data on microcirculatory effect or organ perfusion; data acquisition only under general anesthesia; and no data on the systemic inflammatory response. Our primary intent was to study the effect of fluid resuscitation rate on blood loss and hemoglobin in our hemorrhage model. With respect to our porcine hemorrhage model, there are controversial aspects of these models which were not intended targets of this study, including the necessity of routine pre-injury splenectomy,[41, 42] the type of resuscitation fluid to employ,[39, 43] and the relative advantages of open vs. closed injury mechanisms.[20, 21, 26, 29, 44] With respect to the choice of fluid resuscitation, it is important to note the recent results of the Prehospital Air Medical Plasma (PAMPer) trial,[37] which found that prehospital use of plasma for resuscitation improved survival in trauma patients at risk for hemorrhagic shock. However, we elected to use crystalloid as the resuscitation fluid in our study because we wanted to mimic austere battlefield conditions.

## Conclusions

In a porcine model of uncontrolled intraabdominal hemorrhage, a relatively slow rate (20 mL/min) of intravenous crystalloid resuscitation produced a less negative outcome (decreased blood loss and a smaller decrease in hemoglobin) compared to a more rapid rate (150 mL/min). The study was not powered to determine if there was a difference in short-term survival.

## Supporting information

S1 Fig Statistical analysis. (PDF)

S2 Fig Kaplan meier plot. Survival of injured porcine subjects treated with rapid *vs*. slow crystalloid resuscitation.
(PDF)

S1 Table ARRIVE information. (PDF)

S2 Table Raw data. All raw data from this study, Excel format.
(XLS)
