# Supplementary material for: Fluid administration rate for uncontrolled intraabdominal hemorrhage in swine
Source: PLoS One. 2018 Nov 29;13(11):e0207708. doi: 10.1371/journal.pone.0207708 (PMC6264836; doi:10.1371/journal.pone.0207708)
Supplement: S1 Fig — (PDF) [file pone.0207708.s001.pdf]

INTRODUCTION

Data were received for a study which compared two groups of pigs defined by the type of fluid resuscitation ("rapid" vs. "slow) received in a hemorrhage model. Data consist of 3 to 5 items related to four general categories: coagulation, blood gases, hematology, and vital signs. Other descriptive data were collected as well. The four categories of data items were measured at three time points, baseline, 15 minutes, and either 60 or 180 minutes depending on the group.

Statistical Analysis

For each variable non-paired comparison (i.e., rapid vs. slow) were made at each time point. The two groups are not exactly comparable, because the rapid group had a post-injury observation period of 60 min while the slow group had an observation period of 180 min. The variables affected by this difference include post-injury blood loss and survival.

From a total of 12 pigs subjects assigned to one of two groups (between factor), up to three data points (within factor is time) were measured. The data were examined for outliers, and with a couple of exceptions noted in the results section, they are not visibly present or of a concern for most data items and so non-parametric tests are not necessary. With continuous response variables the relevant statistical analysis here is a normal residual repeated measures ANOVA model which consists of one between subjects factor Group (Rigid/Slow) with data collected at three time points (1-Baseline, 2=15 minutes / 3=60 or 180 minutes); these two factors were evaluated as having fixed effects (that is, no other levels of the factor were used). Their main effects and interaction were evaluated with type III tests of statistical significance. A special type of covariance matrix to model correlations over time was utilized, known as the ante-dependence model at lag 1, chosen over other types of structures based on the type that generally gave the smallest fit statistics (AICC and BIC). The interaction of group and time was evaluated with the group*time pvalue printed in the tables and on the graphs, along with comparisons of the two group means including their differences at each time point with an adjusted pvalue. Plots of the means with confidence intervals are provided, but more importantly also of the differences are provided with the adjusted pvalue printed on the graph for each mean difference. A limitation of the comparisons are that comparisons of the two groups made at time point 3 are based on measurements at 60 minutes for the pigs placed in the “Rapid” level and 180 minutes for the “Slow” level.

Missing data was also a feature of some items, especially those collected at the third time point. In some cases they are due to a measurement “failed” reason, which for some cases of coagulation the following default values were entered (which came from the clinical laboratory literature):

QFA = 20
PT = 37
INR = 5

The amount missing data for other reasons was quite small. Implementation of missing data imputation techniques or other fill in methods were attempted, but the small sample size of 24 pigs did not allow them to produce usable data. The repeated measures ANOVA model works well with small amounts of missing data (i.e., it uses all the data) and is for this study is assumed to not have a noticeable impact on the results.

All statistical significance tests were two-sided. Statistical computations were generated with PROCs GLIMMIX or LIFETEST from SAS/STAT software, version 9.4 (© 2002 - 2012) of the SAS System for Windows (Cary, NC). Graphs were made with SAS SG plotting procedures, including means and differences displayed side-by-side with templates designed to display means at three time points and their differences and also for two-sample tests of means.

Statistical Analysis (Yanala et al.)

Means for the descriptive data items are evaluated first on the next 3 pages.

T-Test for Pre injury blood loss (mL)) vs Group

T-Test Summary	N	Median	Mean/_- Diff	Std Error	Lower Limit	Upper Limit	_T_	df	Pvalue TTest	Pvalue Wlcxn
Rapid	12	381.0	430.5	43.5	334.7	526.2				
Slow	12	425.7	413.8	13.9	383.1	444.5				
Diff (1-2)			16.7	45.7	-81.8	115.2	0.37	13.2	0.72	0.32

There is a large outlier in the Rapid group close to 850, which is considerably larger than all other data values in both groups. Even with this value included, both pvalues from the unequal variance t-test and the non-parametric test indicate not difference between the groups.

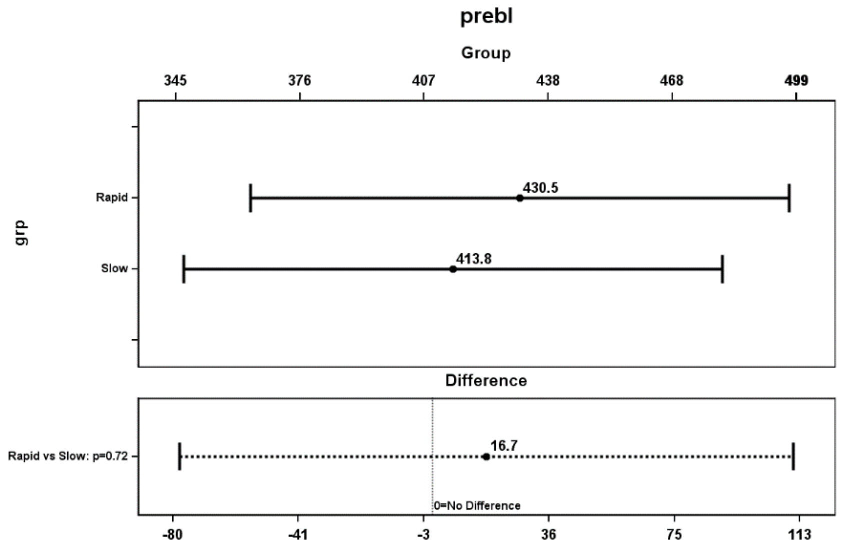

T-Test for Post injury blood loss (mL) vs Group with

T-Test Summary	N	Median	Mean/_- Diff	Std Error	Lower Limit	Upper Limit	_T_	df	Pvalue TTest	Pvalue Wlcxn
Rapid	12	2613.3	2737.5	200.0	2297.3	3177.7				
Slow	12	1506.2	1600.2	104.1	1371.1	1829.2				
Diff (1-2)			1137.4	225.5	660.7	1614.0	5.04	16.5	<.001 **	<.001 **

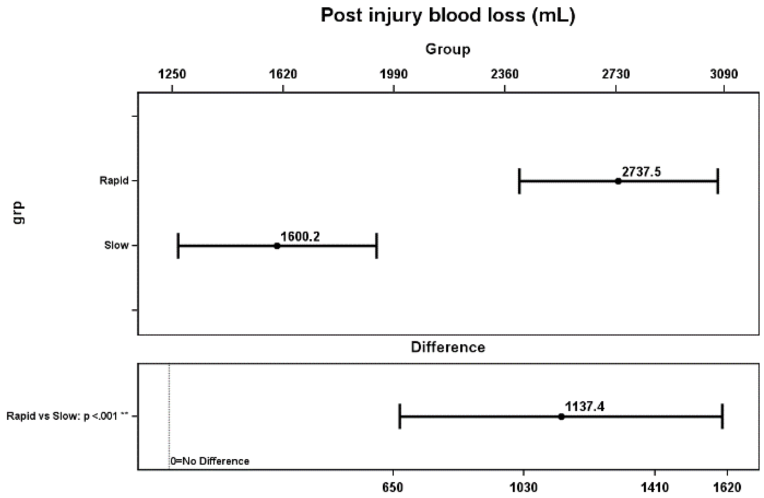

Statistical Analysis (Yanala et al.)

T-Test for Pre injury IV Fluid (mL) vs Group

T-Test Summary	N	Median	Mean/_-Diff	Std Error	Lower Limit	Upper Limit	_T_	df	Pvalue TTest	Pvalue Wlcxn
Rapid	12	1145.0	1288.5	132.3	997.2	1579.8				
Slow	12	1139.3	1138.0	52.3	1022.9	1253.1				
Diff (1-2)			150.5	142.3	-154.0	454.9	1.06	14.4	0.31	0.71

There is a large outlier in the Rapid group well over 2500, which is considerably larger than all other data values in both groups. Even with this value included, both pvalues from the unequal variance t-test and the non-parametric test indicate no difference between the groups.

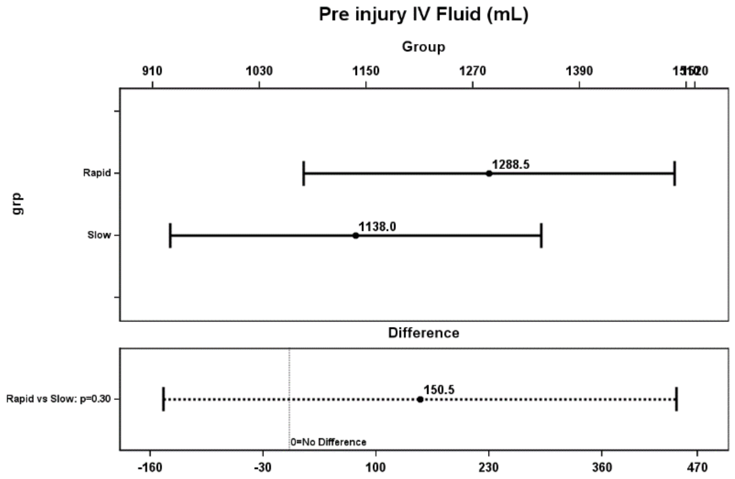

T-Test for Post injury IV Fluid (mL) vs Group

T-Test Summary	N	Nms	Median	Mean/_-Diff	Std Error	Lower Limit	Upper Limit	_T_	df	Pvalue TTest	Pvalue Wlcxn
Rapid	12	0	3625.0	3644.2	150.9	3312.1	3976.2				
Slow	12	0	3720.0	3081.7	496.6	1988.7	4174.6				
Diff (1-2)				562.5	519.0	-558.6	1683.6	1.08	13.0	0.30	0.84

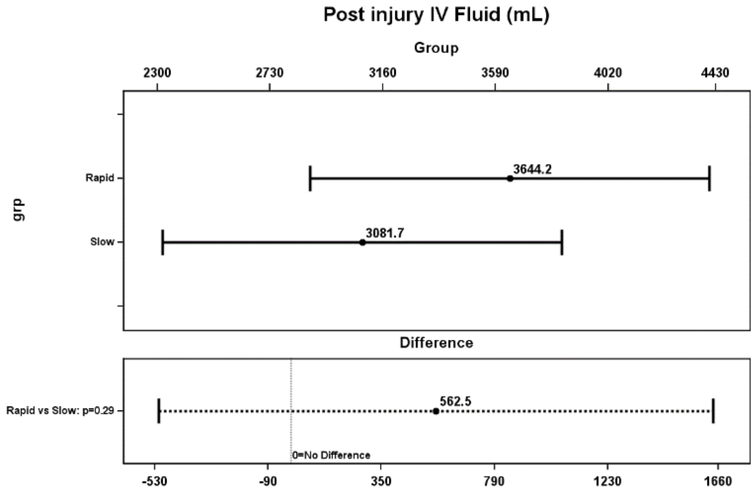

T-Test for Liver Weight (g) vs Group

T-Test Summary	N	Median	Mean/_-Diff	Std Error	Lower Limit	Upper Limit	_T_	df	Pvalue TTest	Pvalue Wlcxn
Rapid	12	822.5	852.9	33.1	779.9	925.8				
Slow	12	924.0	941.0	36.7	860.2	1021.9				
Diff (1-2)			-88.1	49.5	-190.8	14.5	-1.78	22.0	0.089	0.102

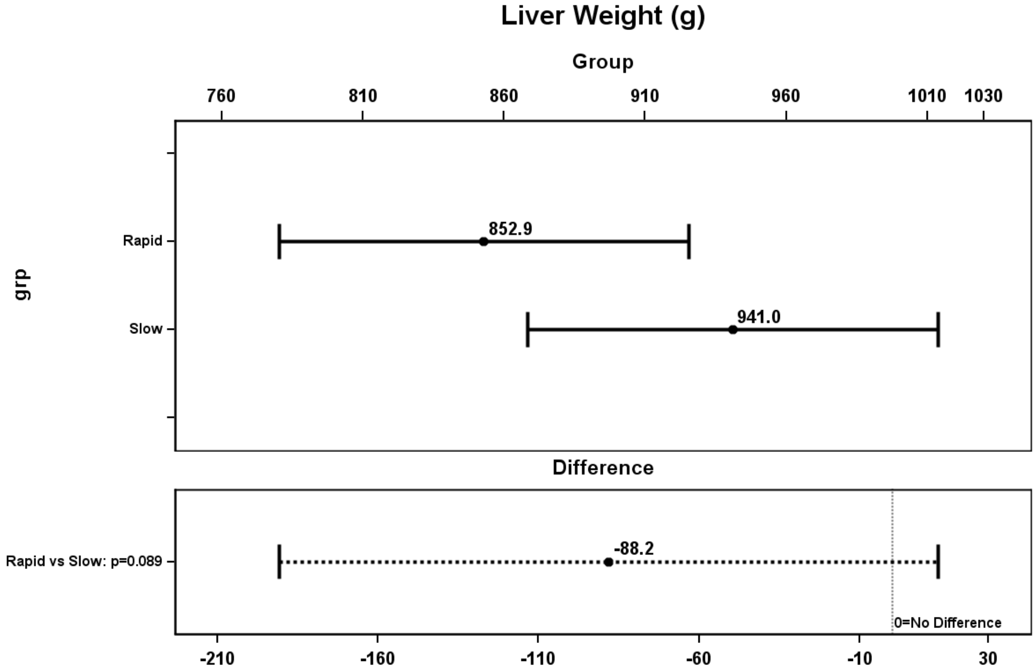

Survival

The observation period for the rapid infusion group was 60 min with a survival rate of 58% (7 out of 12). The observation period for the slow infusion group was 180 minutes with a survival rate of 67% (8 out of 12). The log rank test comparing the survival rate of the two groups was not significant (p=.67). The sample size of 12 and small number of deaths in each group is too small to detect a meaningful difference in the two groups.

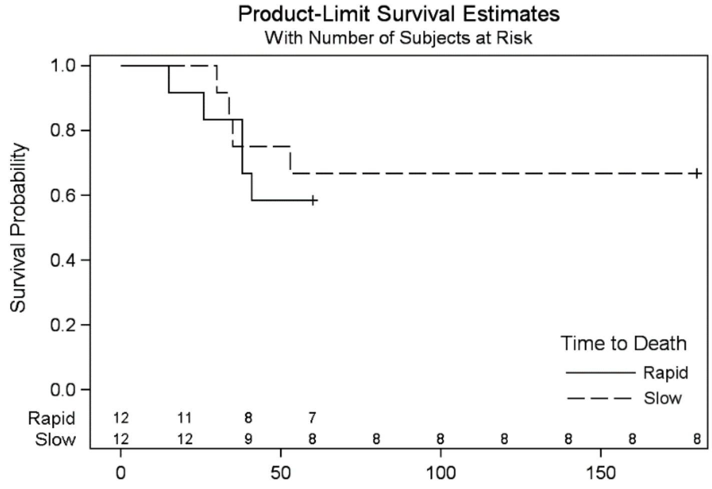

Statistical Analysis (Yanala et al.)

For the various categories of data collected over time, the results are summarized in both tables of means and differences and also graphs that visually display the relationships of the means and also significance of mean differences. Relevant ANOVA Pvalues for the model (ProbF) and group differences are displayed in the tables and on the graphs.

Coagulation

	time	Group	ProbF	Mean	Std Err Mean	Diff	Std Err Diff	Adjp
qfa	1	Rapid	<.001 **	115.25	4.41			
		Slow		108.67	4.41	6.58	6.24	0.58
	2	Rapid		40.83	3.19			
		Slow		63.33	3.33	-22.50	4.61	<.001 **
	3	Rapid		37.25	3.86			
		Slow		46.58	4.31	-9.33	5.78	0.27
pt	1	Rapid	0.002 *	11.48	0.19			
		Slow		10.92	0.19	0.55	0.26	0.107
	2	Rapid		16.62	1.01			
		Slow		11.32	1.03	5.30	1.44	0.003 *
	3	Rapid		18.91	2.51			
		Slow		14.44	2.74	4.47	3.72	0.46
inr	1	Rapid	0.017 *	1.04	0.02			
		Slow		0.91	0.02	0.13	0.03	<.001 **
	2	Rapid		1.51	0.10			
		Slow		0.99	0.10	0.52	0.14	0.004 *
	3	Rapid		1.99	0.39			
		Slow		1.45	0.44	0.54	0.59	0.69

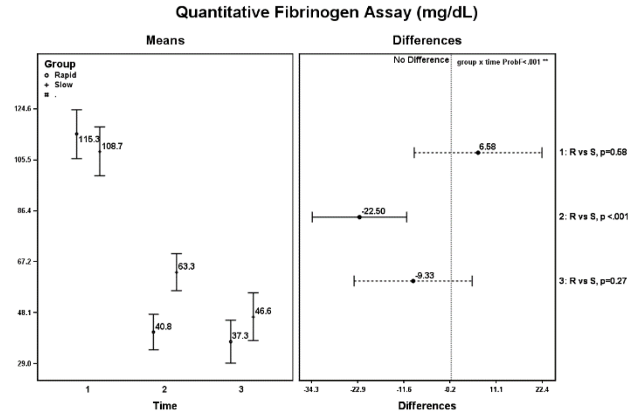

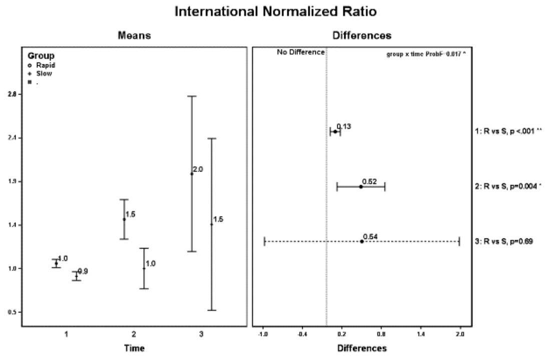

All three coagulation variables exhibit significant differences of rapid versus slow at time 2 (15 minutes). The difference for INR at time 1 (baseline) is perhaps not as impressive since the variability tends to be smaller.

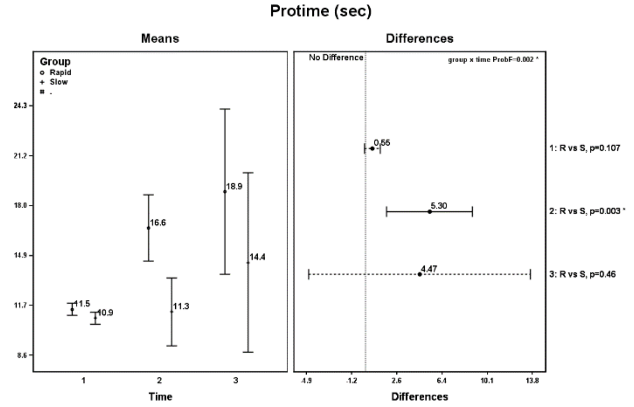

Statistical Analysis (Yanala et al.)

Hematology

vr	tme	Group	ProbF	Mean	Std Err Mean	Diff	Std Err Diff	Adjp
hb	1	Rapid	<.001 **	12.16	0.25			
		Slow		11.67	0.25	0.49	0.35	0.39
	2	Rapid		4.55	0.47			
		Slow		8.39	0.47	-3.84	0.67	<.001 **
	3	Rapid		3.41	0.62			
		Slow		5.96	0.65	-2.55	0.90	0.025 *
hct	1	Rapid	<.001 **	39.48	0.75			
		Slow		39.28	0.75	0.20	1.07	0.99
	2	Rapid		15.66	1.60			
		Slow		28.25	1.60	-12.59	2.26	<.001 **
	3	Rapid		11.81	2.06			
		Slow		20.44	2.15	-8.63	2.98	0.021 *
pltl	1	Rapid	0.40	306.50	23.64			
		Slow		314.75	23.64	-8.25	33.44	0.99
	2	Rapid		146.67	17.43			
		Slow		215.17	17.43	-68.50	24.65	0.029 *
	3	Rapid		122.33	19.13			
		Slow		170.62	20.56	-48.29	28.08	0.23

Hemoglobin (g/dL)

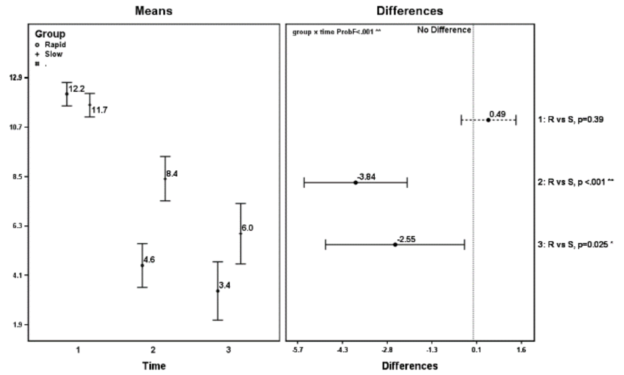

Platelets (1,000/μL)

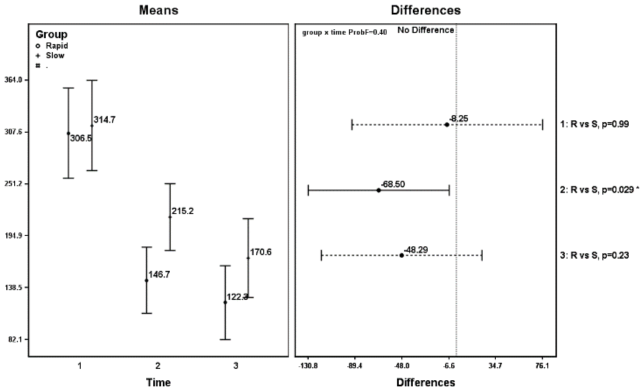

Hematocrit (%)

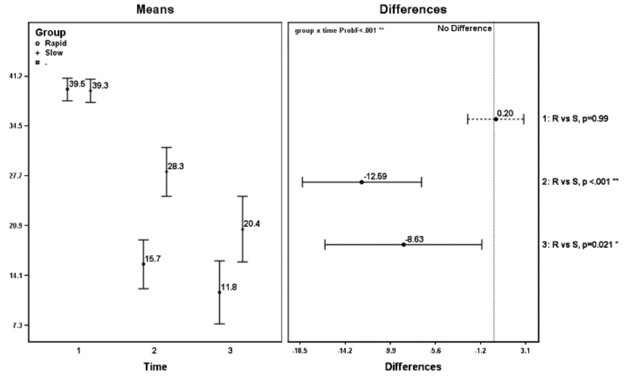

Differences between Rapid and Slow are largest at time 2 for all three Hematology variables.

Statistical Analysis (Yanala et al.)

Blood Gases

vr	tme	Group	ProbF	Mean	Std Err Mean	Diff	Std Err Diff	Adjp
pH	1	Rapid	0.23	7.46	0.02			
		Slow		7.44	0.02	0.02	0.03	0.95
	2	Rapid		7.40	0.03			
		Slow		7.44	0.03	-0.03	0.04	0.80
	3	Rapid		7.45	0.05			
		Slow		7.37	0.04	0.08	0.06	0.52
pC02	1	Rapid	0.20	39.33	2.78			
		Slow		46.50	2.78	-7.18	3.93	0.20
	2	Rapid		36.50	3.41			
		Slow		34.43	3.41	2.07	4.82	0.96
	3	Rapid		31.09	4.81			
		Slow		34.63	4.64	-3.53	6.68	0.92
p02	1	Rapid	0.62	443.25	8.83			
		Slow		450.88	8.83	-7.63	12.49	0.90
	2	Rapid		445.94	13.01			
		Slow		434.48	12.41	11.47	17.98	0.89
	3	Rapid		412.95	15.69			
		Slow		414.37	14.96	-1.42	21.68	1.00

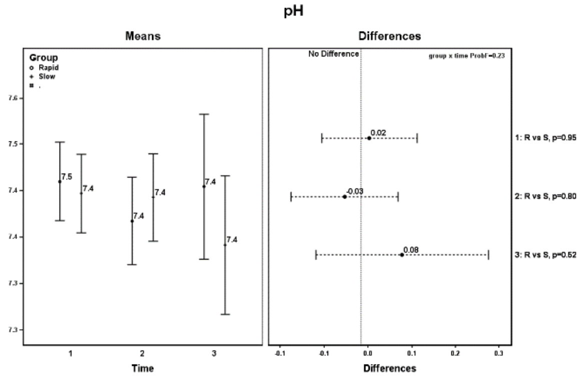

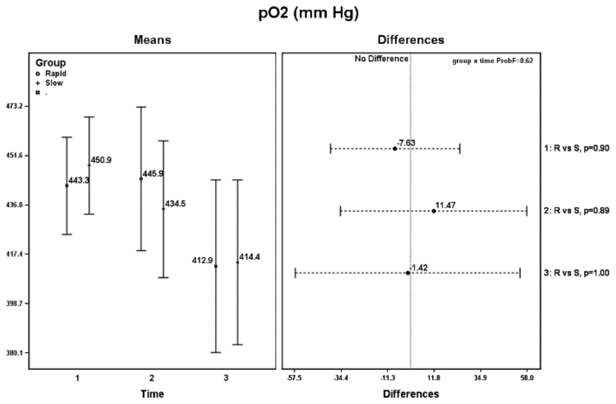

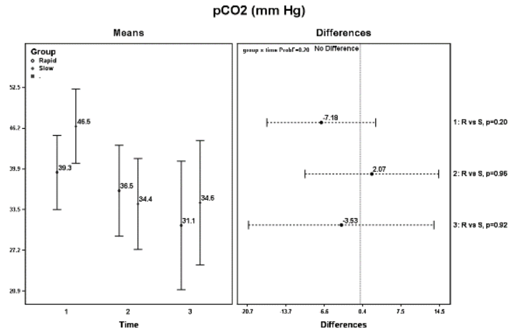

No significant differences are observed for the three Blood Gas variables.

Blood Gases

vr	tme	Group	ProbF	Mean	Std Err Mean	Diff	Std Err Diff	Adjp
Bxcs	1	Rapid	0.30	3.36	0.74			
		Slow		4.43	0.74	-1.08	1.04	0.62
	2	Rapid		-2.76	0.98			
		Slow		-1.70	0.98	-1.06	1.38	0.79
	3	Rapid		-4.27	1.99			
		Slow		-6.40	1.94	2.13	2.78	0.79
Bicarb	1	Rapid	0.51	27.02	0.69			
		Slow		28.73	0.69	-1.71	0.98	0.22
	2	Rapid		21.53	1.12			
		Slow		21.81	1.12	-0.28	1.58	1.00
	3	Rapid		19.79	1.92			
		Slow		18.34	1.89	1.45	2.70	0.90

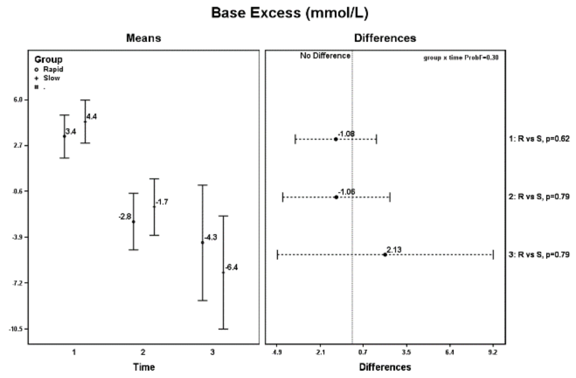

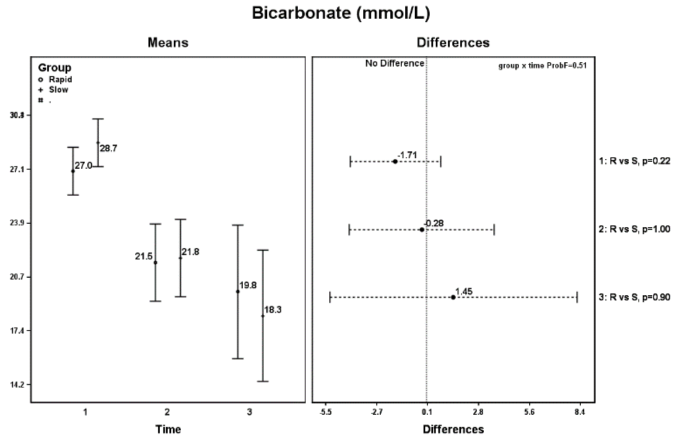

No significant differences exist for these two blood gas variables.

Vital Signs

vr	tme	Group	ProbF	Mean	Std Err Mean	Diff	Std Err Diff	Adjp
map	1	Rapid	0.007 *	112.83	4.56			
		Slow		105.08	4.56	7.75	6.45	0.52
	2	Rapid		54.67	7.22			
		Slow		32.83	7.22	21.83	10.21	0.11
	3	Rapid		26.58	7.02			
		Slow		39.08	7.02	-12.50	9.93	0.48
hr	1	Rapid	0.077	106.75	5.20			
		Slow		90.00	5.20	16.75	7.35	0.090
	2	Rapid		108.17	6.90			
		Slow		110.50	6.90	-2.33	9.76	0.99
	3	Rapid		99.50	9.10			
		Slow		112.17	9.10	-12.67	12.87	0.69
temp	1	Rapid	0.16	36.87	0.26			
		Slow		36.30	0.26	0.57	0.37	0.23
	2	Rapid		36.51	0.23			
		Slow		35.93	0.23	0.58	0.32	0.15
	3	Rapid		36.06	0.23			
		Slow		35.06	0.23	1.00	0.32	0.010 *

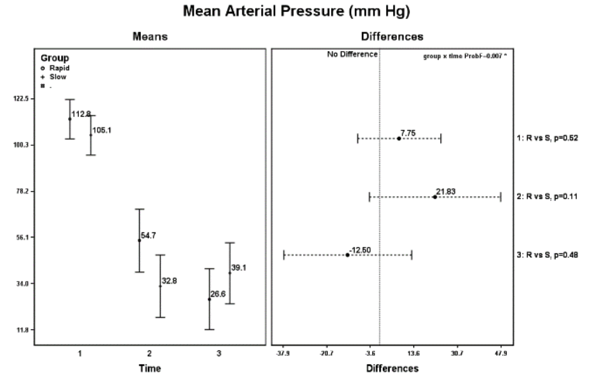

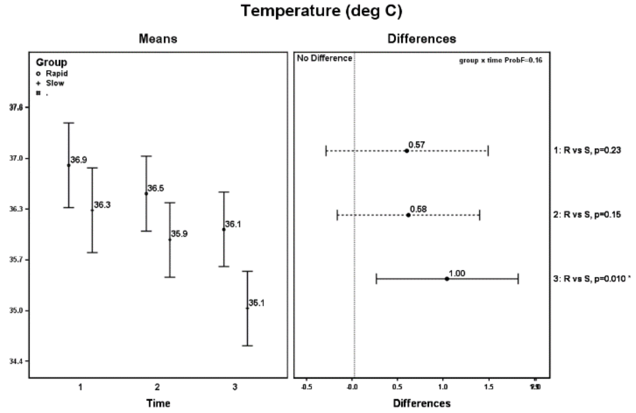

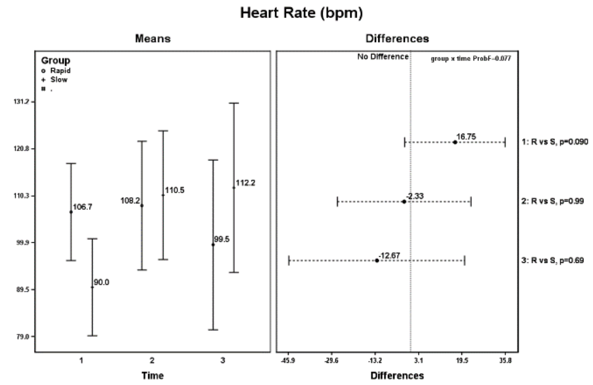

The differences for Vital Signs are not as conclusive. The overall F-test for MAP is significant, but the adjusted differences are not. This feature occasionally occurs, esp. with correlated longitudinal data. Rapid vs. Slow at time 2 has the smallest adjusted pvalue.
