# Supplementary figures and images for: Fluid administration rate for uncontrolled intraabdominal hemorrhage in swine
Source: PLoS One. 2018 Nov 29;13(11):e0207708. doi: 10.1371/journal.pone.0207708 (PMC6264836; doi:10.1371/journal.pone.0207708)

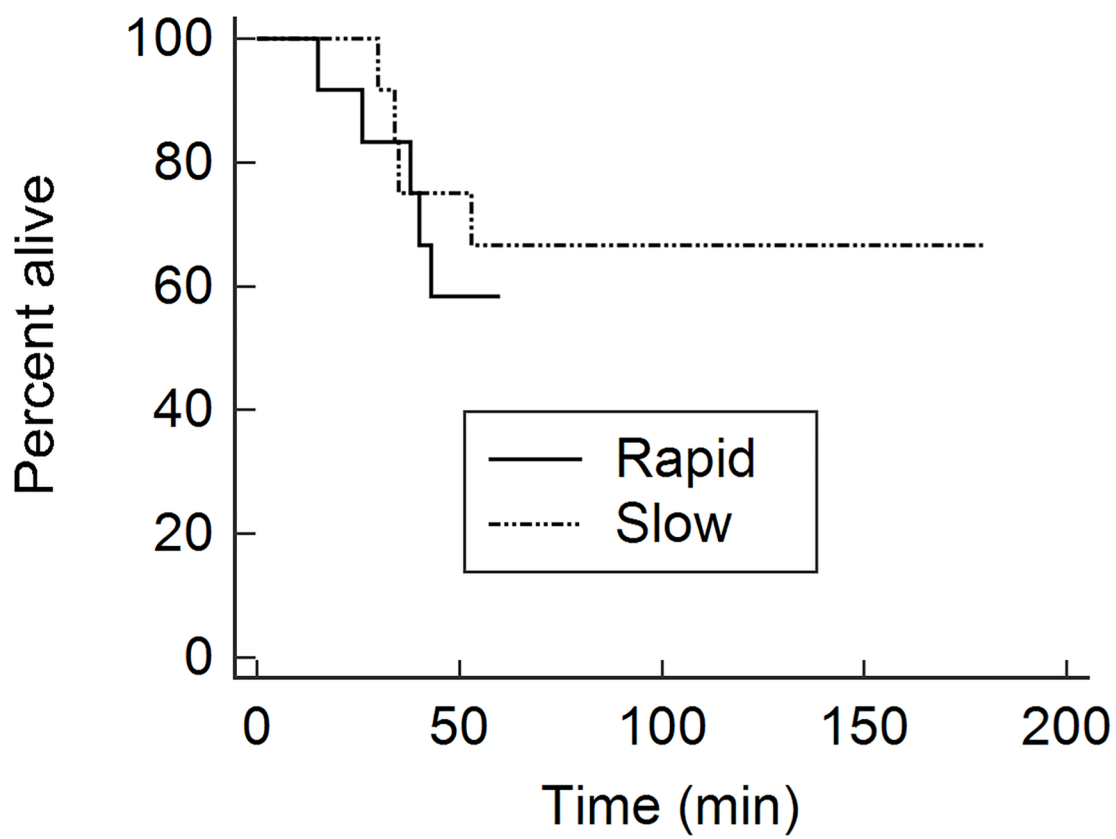

Fig. S1

Supplement: S2 Fig — Survival of injured porcine subjects treated with rapid vs. slow crystalloid resuscitation. (PDF) [file pone.0207708.s002.pdf]
